# The influence of distribution, severity and volume of posttraumatic bone bruise on functional outcome after ACL reconstruction for isolated ACL injuries

**DOI:** 10.1007/s00402-023-04907-w

**Published:** 2023-06-03

**Authors:** Bastian Mester, Patric Kröpil, Tobias Ohmann, Christoph Schleich, Claas Güthoff, Arthur Praetorius, Marcel Dudda, Christian Schoepp

**Affiliations:** 1 Department for Trauma, Hand and Reconstructive Surgery, University Hospital Essen, Hufelandstraße 55, 45147 Essen, Germany; 2 Department for Arthroscopic Surgery, Sports Traumatology and Sports Medicine, BG Klinikum Duisburg, Großenbaumer Allee 250, 47249 Duisburg, Germany; 3 Department for Radiology, BG Klinikum Duisburg, Großenbaumer Allee 250, 47249 Duisburg, Germany; 4 Research Department, BG Klinikum Duisburg, Großenbaumer Allee 250, 47249 Duisburg, Germany; 5 Centre for Clinical Research, BG Klinikum Unfallkrankenhaus Berlin, Warener Straße 7, 12683 Berlin, Germany; 6 Department for Diagnostic and Interventional Radiology, University Hospital Düsseldorf, Moorenstraße 5, 40225 Düsseldorf, Germany; 7 Department for Orthopedics and Trauma Surgery, BG Klinikum Duisburg, Großenbaumer Allee 250, 47249 Duisburg, Germany

**Keywords:** ACL injury, Bone bruise, Bone marrow edema, MRI, Outcome

## Abstract

**Introduction:**

Posttraumatic MRI of ACL tears show a high prevalence of bone bruise (BB) without macroscopic proof of chondral damage. Controversial results are described concerning the association between BB and outcome after ACL tear. Aim of this study is to evaluate the influence of distribution, severity and volume of BB in isolated ACL injuries on function, quality of life and muscle strength following ACL reconstruction (ACLR).

**Materials and Methods:**

MRI of n = 122 patients treated by ACLR without concomitant pathologies were evaluated. BB was differentiated by four localizations: medial/lateral femoral condyle (MFC/LFC) and medial/lateral tibial plateau (MTP/LTP). Severity was graded according to Costa-Paz. BB volumes of n = 46 patients were quantified (software-assisted volumetry). Outcome was measured by Lysholm Score (LS), Tegner Activity Scale (TAS), IKDC, isokinetics and SF-36. Measurements were conducted preoperatively (t0), 6 weeks (t1), 26 weeks (t2) and 52 weeks (t3) after ACLR.

**Results:**

The prevalence of BB was 91.8%. LTP was present in 91.8%, LFC 64.8%, MTP 49.2% and MFC 28.7%. 18.9% were classified Costa-Paz I, 58.2% II and 14.8% III. Total BB volume was 21.84 ± 15.27 cm^3^, the highest value for LTP (14.31 ± 9.93 cm^3^). LS/TAS/IKDC/SF-36/isokinetics improved significantly between t0–t3 (p < 0.001). Distribution, severity and volume had no influence on LS/TAS/IKDC/SF-36/isokinetics (n.s.).

**Conclusions:**

No impact of BB after ACLR on function, quality of life and objective muscle strength was shown, unaffected by concomitant pathologies. Previous data regarding prevalence and distribution is confirmed. These results help surgeons counselling patients regarding the interpretation of extensive BB findings. Long-time follow-up studies are mandatory to evaluate an impact of BB on knee function due to secondary arthritis.

## Introduction

Large external forces in anterior cruciate ligament (ACL) injury mechanisms cause a traumatic impact between femoral and tibial joint surface [1–3]. Subsequently, posttraumatic Magnetic Resonance Imaging (MRI) shows a high prevalence of traumatic bone bruise (BB) with low T1-weighted and high T2-weighted and PD-weighted signal intensity [4]. According to literature, the overall prevalence of BB after ACL injury is reported between 78 and 100% [1, 5–8].

Concomitant injuries like meniscal tears and collateral ligament lesions are frequently reported in the context of ACL injuries. Although ACL tears are regularly associated with traumatic BB, there is no initial arthroscopic proof of macroscopic damage of the overlying cartilage during ACL reconstruction (ACLR) in most cases. Histologically, BB is characterized by microtrabecular fractures, accompanied by chondrocyte death [9]. These findings imply subclinical chondral defects to the overlying cartilage [10].

Although there has been some research effort concerning the influence of posttraumatic BB in ACL injuries, its functional significance remains controversial. Some authors could not find any influence of posttraumatic BB in functional outcome scores and quality of life after ACL injury and following ACLR [6–8, 11–15]. Other studies show inferior results in terms of higher pain levels and increased disability, depending on prevalence, distribution pattern and volume of posttraumatic BB [1, 9, 16–20]. According to the very few studies reporting results in the mid- to long-term follow-up, BB seems not to affect the clinical outcome after ACLR [13]. Though, on a morphologic point of view BB can be associated with initial chondral damage as well as subchondral injury with surface impression that is detectable by MRI assessment, leading to progression in cartilage degradation in the mid- to long-term follow-up [20, 21].

There are very limited data about the outcome following ACLR for ACL tears without relevant concomitant injuries, as in available studies meniscal tears, collateral/posterior cruciate ligament tears, cartilage lesions have not been excluded systematically. Thus, the implication of BB in isolated ACL tears has not been evaluated sufficiently in the past. Aim of this study is to evaluate the influence of distribution, severity and volume of BB in isolated ACL injuries on the functional outcome, quality of life and muscle strength following ACLR within the first year after surgery. To our knowledge, this is the first study evaluating the impact of BB characteristics, including software-assisted volumetry data, on functional short-term outcome parameters, health-related quality of life and—uniquely—objective isokinetic muscle performance at once. Thereby, the focus lies on isolated ACL tears by systematic exclusion of accompanying knee injuries.

## Materials and methods

For this study, demographic and clinical patient data were recorded prospectively from a cohort of ACL-deficient patients who underwent ACLR as part of a prospective randomized trial (German Clinical Trials Register number 00011774). A total of n = 145 patients with isolated ACL injuries and primary ACLR was identified.

Ethical approval was obtained from the ethical committee of the University of Witten Herdecke, Germany (No. 14_2015). The informed consent was given by each patient included in this study.

All patients underwent primary arthroscopic ACLR between 06/2016 and 04/2019. In all cases, graft choice was ipsilateral hamstrings. All ACLR were performed following a standardized procedure protocol by two experienced arthroscopic surgeons at one institution (single centre study).

Revision ACLR were excluded as well as relevant concomitant intraarticular pathologies, ruled out by MRI, clinical examination as well as diagnostic arthroscopy: meniscal tears suitable for suture repair, collateral/posterior cruciate ligament injuries, cartilage lesions requiring cartilage repair (ICRS stages 3 and 4). Patients with pre-existent rheumatoid arthritis and osteoarthritis or history of previous knee trauma or surgery (ACL repair, meniscal repair) have also been excluded.

As proposed in previous research evaluating the influence of BB on knee function in ACL-injured patients [16], the maximum interval between injury and MRI was set to 12 weeks. The targeted interval between injury and ACLR was 52 weeks.

N = 145 patients were initially assessed for eligibility. In n = 16 cases the interval between injury and MRI exceeded twelve weeks, for n = 6 MRI data were not accessible anymore and n = 1 withdrew the consent after inclusion. These n = 23 patients were excluded. Thus, n = 122 patients (42 female, 80 male; mean age 32.78 ± 11.40 years) were included for descriptive data analysis. Explorative analysis of the influence of BB characteristics on outcome measures was conducted for all patients presenting with BB in at least one localization (n = 112). Additionally, statistical testing was performed for comparative reasons for the n = 10 patients with no proof of BB.

**Fig. 1:**
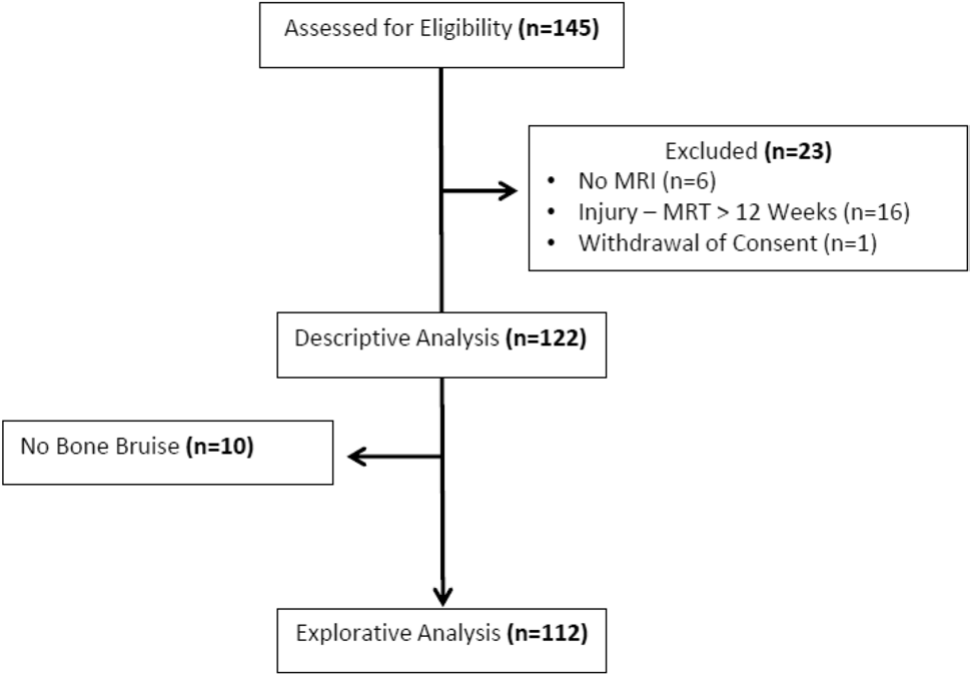
Flow of patients through the study. CONSORT diagram presenting the enrolment of the final study population according to [22]

The patient’s enrolment process and application of inclusion and exclusion criteria is shown in Fig. 1 [22].

Two independent and blinded researchers (one surgeon and one radiologist) evaluated posttraumatic digital MRI regarding prevalence, distribution pattern, severity and volume of BB. In favour of comparable investigation conditions, MRI of all patients included met basic standard requirements (digital T2-weighted and PD sequences in the coronal and sagittal plane).

If prevalent, BB was differentiated by four localizations: medial/lateral femoral condyle and medial/lateral tibial plateau (MFC/LFC/MTP/LTP). The different distribution patterns were registered. Due to clinical experience and to results of the available literature BB patterns were categorized into a more common lateral distribution (LFC and/or LTP) versus a less common (additional) medial distribution (MFC and/or MTP).

In a subgroup of n = 46 patients whose MRI data was applicable, BB volume (cm^3^) was quantified semi-automatically via software-assisted volumetry (syngo via VB 30 A; Siemens Healthineers, Erlangen, Germany). Software-assisted volumetry has been shown to be an accurate tool to measure volumes in MRI imaging [23] and can be regarded a reliable alternative to manual measuring techniques that have been utilized in the past [7]. The volume was measured separately in MFC/LFC/MTP/LTP. The total BB volume was the sum of the single volumes in the four localizations.

**Fig. 2:**
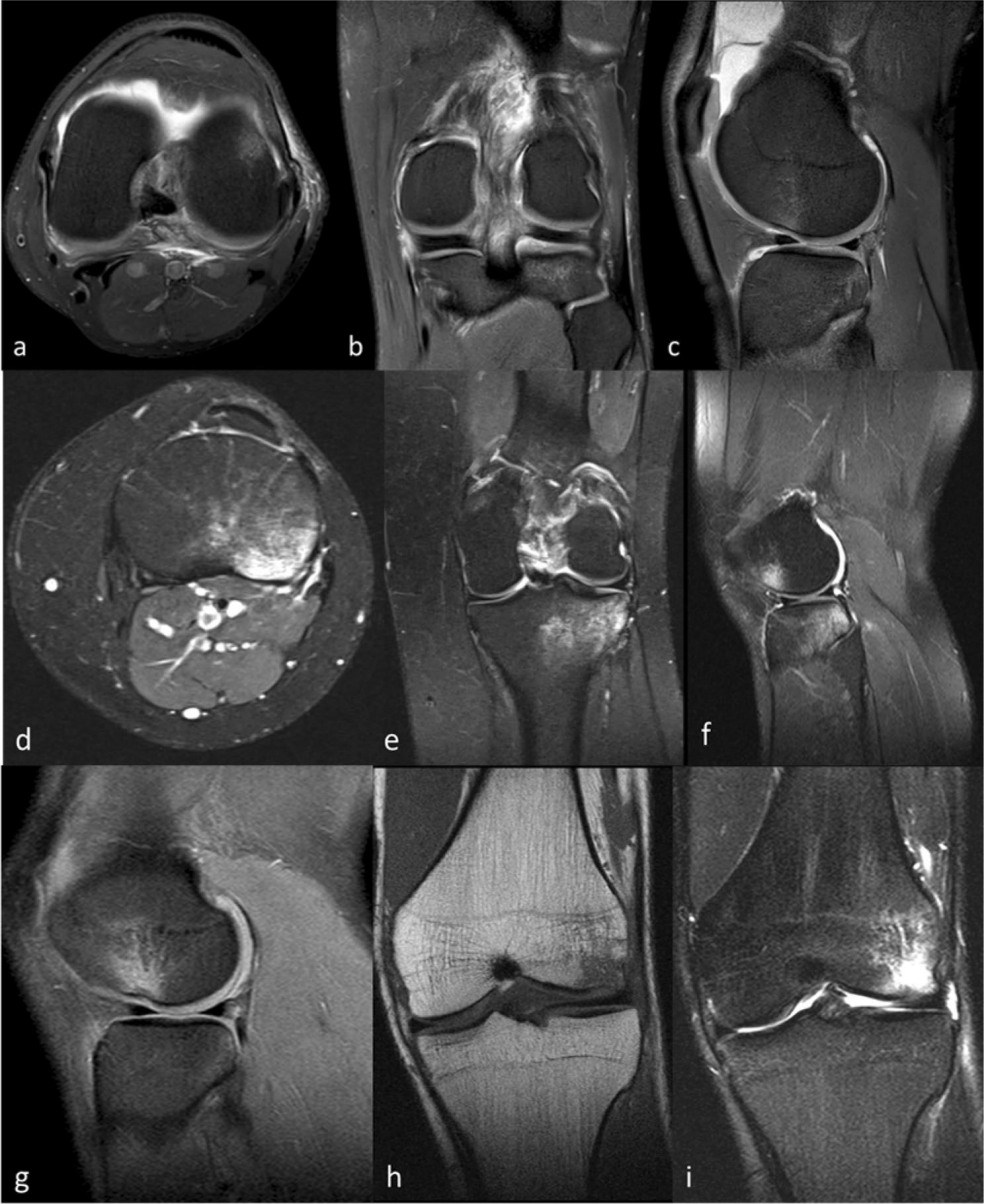
MRI examples of study patients presenting with posttraumatic BB accompanying ACL injury. **a**–**c** Costa-Paz type 1 of the lateral tibial plateau and lateral femoral condyle; axial, coronal and sagittal. **d**–**f** BB type Costa-Paz type 2 of the lateral tibial plateau and lateral femoral condyle; axial, coronal and sagittal. **g**–**i** Costa-Paz type 3 of the lateral femoral condyle with “lateral femoral notch sign”; sagittal and coronal. (**a**–**g**, **i** = PD tse, **h** = T1)

The severity of BB was classified according to the grading system published by Costa-Paz et al. into type I–III [24]. Type I is defined as diffuse signal, often reticular and distant from the articular surface (Fig. 2a–c). A localized signal bordering the articular surface is considered a type II (Fig. 2d–f). Type III is described as a disruption or depression of the normal contour of the cortical surface [7] (Fig. 2g–i). In cases with presence of BB in more than one localization, the most severe finding according to Costa-Paz was stated as synopsis.

Type 3 was further differentiated for the prevalence of a lateral femoral notch sign (LFNS) or an apple-bite fracture (ABF). LFNS is defined as a depression more than 1.5 mm deep in the lateral femoral condyle near the terminal sulcus [25, 26]. ABF is a fracture of the posterolateral tibia plateau caused by a ventral subluxation of the tibia and impact of the femur in the posterolateral tibia plateau [27].

MRI interpretations of both responsible researchers were correlated. The initial consensus rate regarding prevalence, distribution pattern and severity was > 90%. In the few remaining cases showing mismatch, MRI images were re-interpreted conjointly, and a consensus could be achieved 100%.

Demographic characteristics were recorded from the database. Injury mechanisms were described (contact versus non-contact). Intervals between date of injury and MRI and between injury and ACLR were calculated. The demographic data and injury characteristics are presented in Table 1.

**Table 1:** Demographic data and injury characteristics. n = 122

Gender—n (%)	
Female	42 (34.4)
Male	80 (65.6)
Age—mean ± SD (range)	32.78 ± 11.40 years (18–64)
BMI—mean ± SD (range)	25.96 ± 3.64 kg/m^2^ (18.6–35)
Smoking—n (%)	
Yes	32 (26.2)
No	90 (73.8)
ACL-injured leg—n (%)	
Left	65 (53.3)
Right	57 (46.7)
Mechanism—n (%)	
Non-contact	107 (87.7)
Contact	15 (12.3)
Intervals—mean ± SD (range; median)	
Injury—MRI	11.24 ± 13.28 days (0–79; 6)
Injury—ACL reconstruction	93.78 ± 151.01 days (8–1392; 54.5)

Functional outcome was measured using Lysholm Score (LS), Tegner Activity Scale (TAS) and International Knee Documentation Committee IKDC form [28–31]. Health-related quality of life was assessed by completion of the Short-Form 36 (SF-36) questionnaire [32].

Isokinetic strength testing was carried out on an isokinetic test device (Biodex System 3; Biodex Medical Systems, Shirley, USA) for knee extension and flexion. The device is reported to be able to objectively measure torque, power, angular velocity, or muscular endurance capacity [33]. All data was collected and inspected by the same researcher to select maximal effort repetitions for each participant. All knee extension (EXT_max_ [DMM]) and flexion (FLEX_max_ [DMM]) cycles were reported as mean relative peak-torque values.

Functional measurements were conducted preoperatively (t0) and 6 weeks (t1), 26 weeks (t2) and 52 weeks (t3) postoperatively after ACLR. Isokinetic strength testing was performed at t2 and t3.

### Statistical analysis

Standard descriptive statistics were performed with continuous variables reported as means and standard deviations; categorical variables are tabulated as numbers and percentages.

The Mann–Whitney-U-Test or Kruskal–Wallis-Test for independent data and the Friedman-Test for dependent data were applied in skewed distributions. Correlations were calculated using Spearman’s Rho.

To account for multiplicity and an unplanned interim analysis conducted beforehand 0.001 was determined as significance level for all tests. All statistical analyses were performed using SPSS 22.0 (IBM Corp., Armonk, USA) and Stata 14.2.

## Results

The mean interval between ACL injury and MRI was 11.24 ± 13.28 days (min 0, max 79; median 6), the interval between injury and ACLR was 93.78 ± 151.01 days (min 8, max 1392; median 54.5). The interval between injury and MRI in patients without BB in any localization (n = 10) was 25.40 ± 20.52 days (min 7, max 79), in patient with proof of BB (n = 112) 9.97 ± 11.77 days (min 0, max 68). This difference was statistically significant (p = 0.0001). Out of all patients, n = 3 exceeded an interval between injury and operation of 52 weeks (1392/663/522 days). None of these three had a proof of posttraumatic BB.

Regarding the injury mechanisms, predominantly non-contact injuries were reported (n = 107 patients, 87.7%).

The prevalence of BB in at least one localization was 91.8% (n = 112/122). 70.5% of all patients showed a BB involvement of both femur and tibia. There was no case of isolated femoral BB in our collective. Regarding the distribution pattern, LTP was the most common presenting in 91.8, LFC was found in 64.8%. MTP and MFC were found in n = 60 and n = 35 cases. Table 2 gives an overview about BB prevalence and distribution patterns.

**Table 2:** Bone bruise characteristics

Prevalence	
BB prevalent	BB absent
n = 112	n = 10
91.8%	8.2%

Distribution		
Compartment	Frequency (n)	Frequency (%)
LTP	112	91.8
LFC	79	64.8
MTP	60	49.2
MFC	35	28.7

Lateral versus medial distribution	
LTP and/or LFC	MTP and/or MFC
n = 71	n = 41
58.2%	33.6%

Severity			
Costa-Paz I	Costa-Paz II		Costa-Paz III
n = 23	n = 71		n = 18
18.9%	58.2%		14.8%

LFNS	ABF	LFNS + ABF
n = 12	n = 3	n = 2
9.8%	2.5%	1.6%

BB prevalence (sample n = 122); distribution pattern, more common lateral versus less common medial distribution, severity according to Costa-Paz et al. [24] grade I–III

*LTP* lateral tibial plateau, *LFC* lateral femoral condyle, *MTP* medial tibial plateau, *MFC* medial femoral condyle, *LFNS* “Lateral femoral notch sign”, *ABF* “Apple bite fracture”

Distinguishing the severity of posttraumatic BB, n = 23 patients (20.5%) were classified Costa-Paz I, n = 71 (63.4%) Costa-Paz II and n = 18 (16.1%) Costa-Paz III. LFNS was found in n = 12 patients, ABF in n = 3. The combination of LFNS plus ABF was present in n = 2 patients.

In the subgroup of n = 46 patients with additional volumetry data, mean total volume was 21.84 ± 15.27 cm^3^. In accordance with the given sequence of BB localizations, the highest mean volume was measured for LTP with 14.31 ± 9.93 cm^3^ (min 0.73, max 36.32). For LFC the mean volume was 6.57 ± 6.41cm^3^ (min 0.49, max 33.08), for MTP 9.01 ± 7.77 (min 0.63, max 25.43) and for MFC 2.37 ± 1.80 (min 0.44, max 5.97). The total volume of BB did not inversely correlate with the interval between injury and MRI (p > 0.05).

**Fig. 3:**
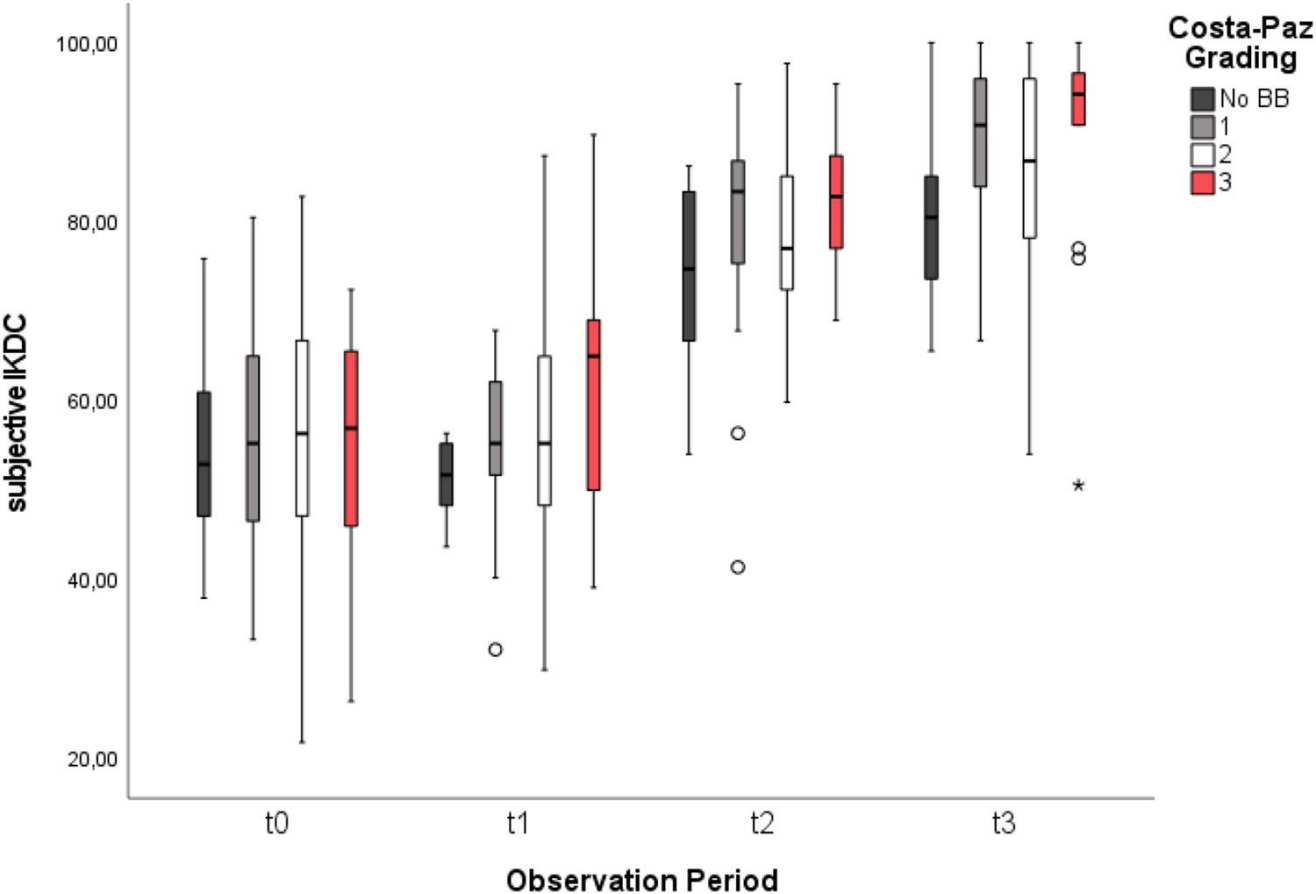
IKDC improvement over time. Mean subjective IKDC Scores and their improvement for all patients presenting with no BB and BB severities Costa-Paz 1–3 preoperatively (t0), 6 weeks (t1), 26 weeks (t2) and 52 weeks (t3) after the operation

**Fig. 4:**
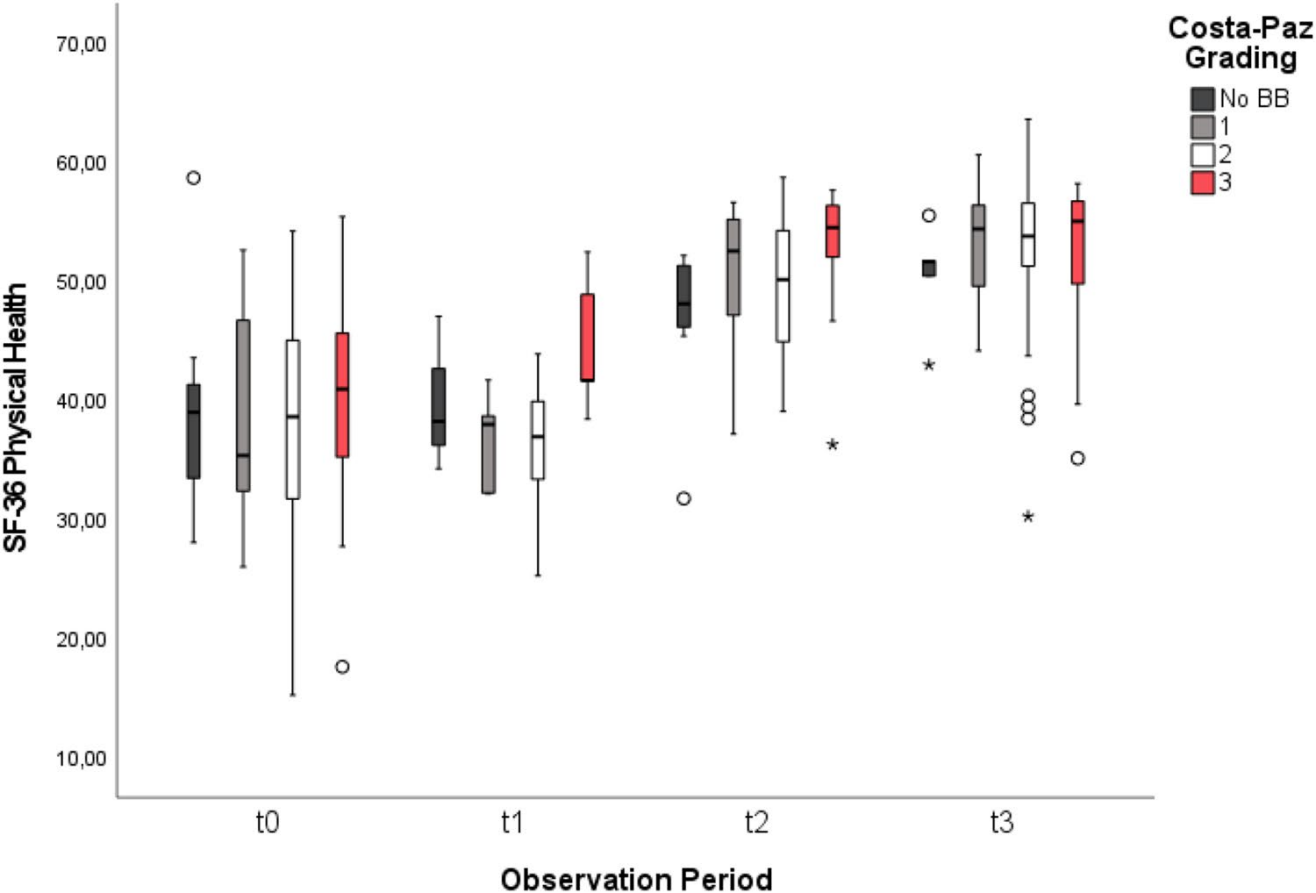
SF-36 improvement over time. Mean SF-36 Physical Health Summary Scales and their improvement for all patients presenting with no BB and BB severities Costa-Paz 1–3 preoperatively (t0), 6 weeks (t1), 26 weeks (t2) and 52 weeks (t3) after the operation

We found a significant improvement for all functional outcome measures in terms of LS (Chi^2^(4) = 194.0, p < 0.001), TAS (Chi^2^(4) = 130.1, p < 0.001) and IKDC (Chi^2^(4) = 240.3, p < 0.001) over time, irrespective of BB characteristics (Fig. 3). There was a significant improvement of quality of life in terms of SF-36 (Chi^2^(4) = 27.6, p < 0.001), shown in Fig. 4.

There was no impact of an additional medial distribution pattern of BB (MFC and/or MTP) on LS, TAS, IKDC and SF-36 compared to a lateral distribution (LFC and/or LTP) at t1–t3 (n.s.). The grading according to Costa-Paz had no influence on LS, TAS, IKDC and SF-36 (n.s.). The total BB volume showed no correlation with LS, TAS, IKDC and SF-36 on any follow-up examination t1–t3 (n.s.).

**Fig. 5:**
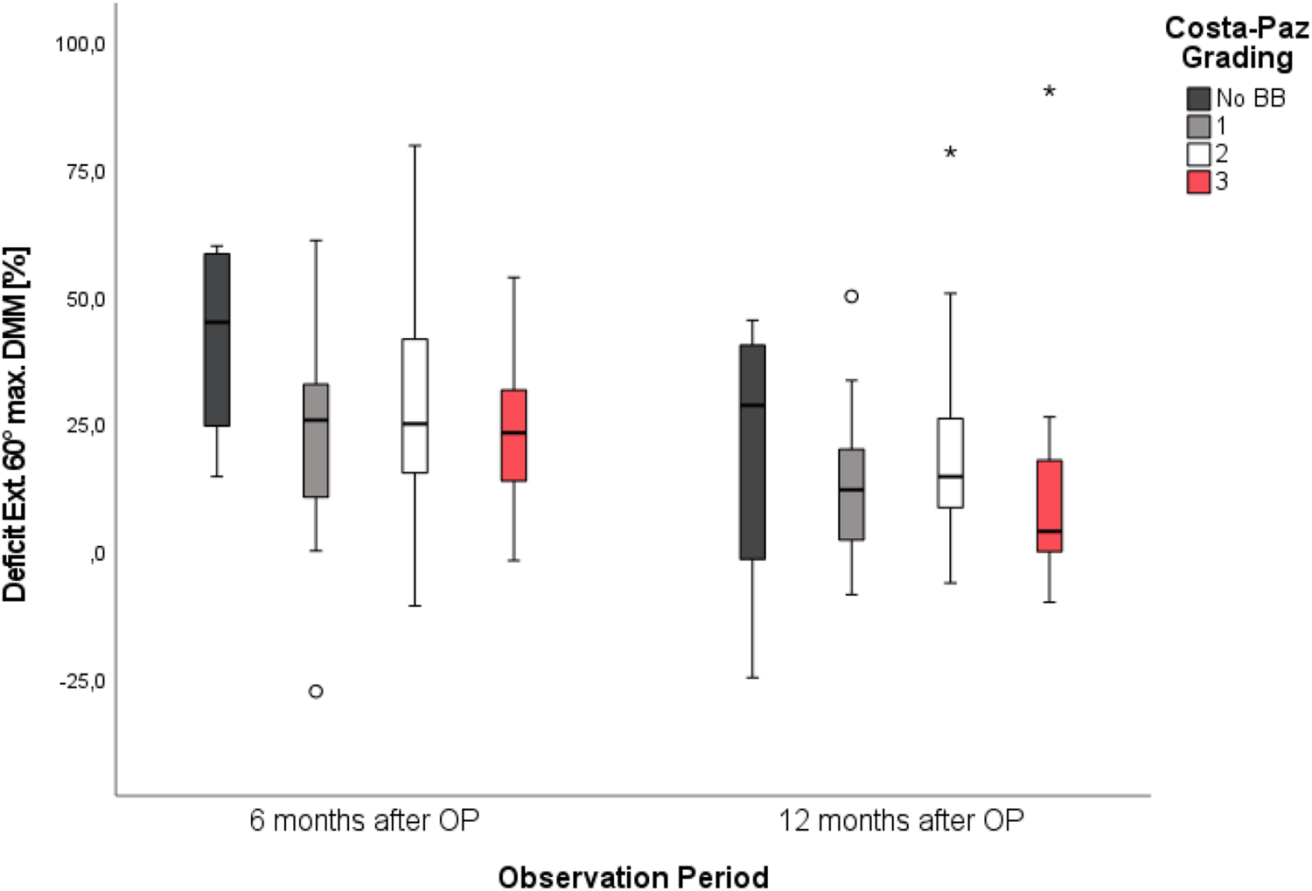
Isokinetic extension strength improvement over time. Reduction of isokinetic strength deficit for knee extension for all patients presenting with no BB and BB severities Costa-Paz 1–3 26 weeks (t2) and 52 weeks (t3) after the operation

**Fig. 6:**
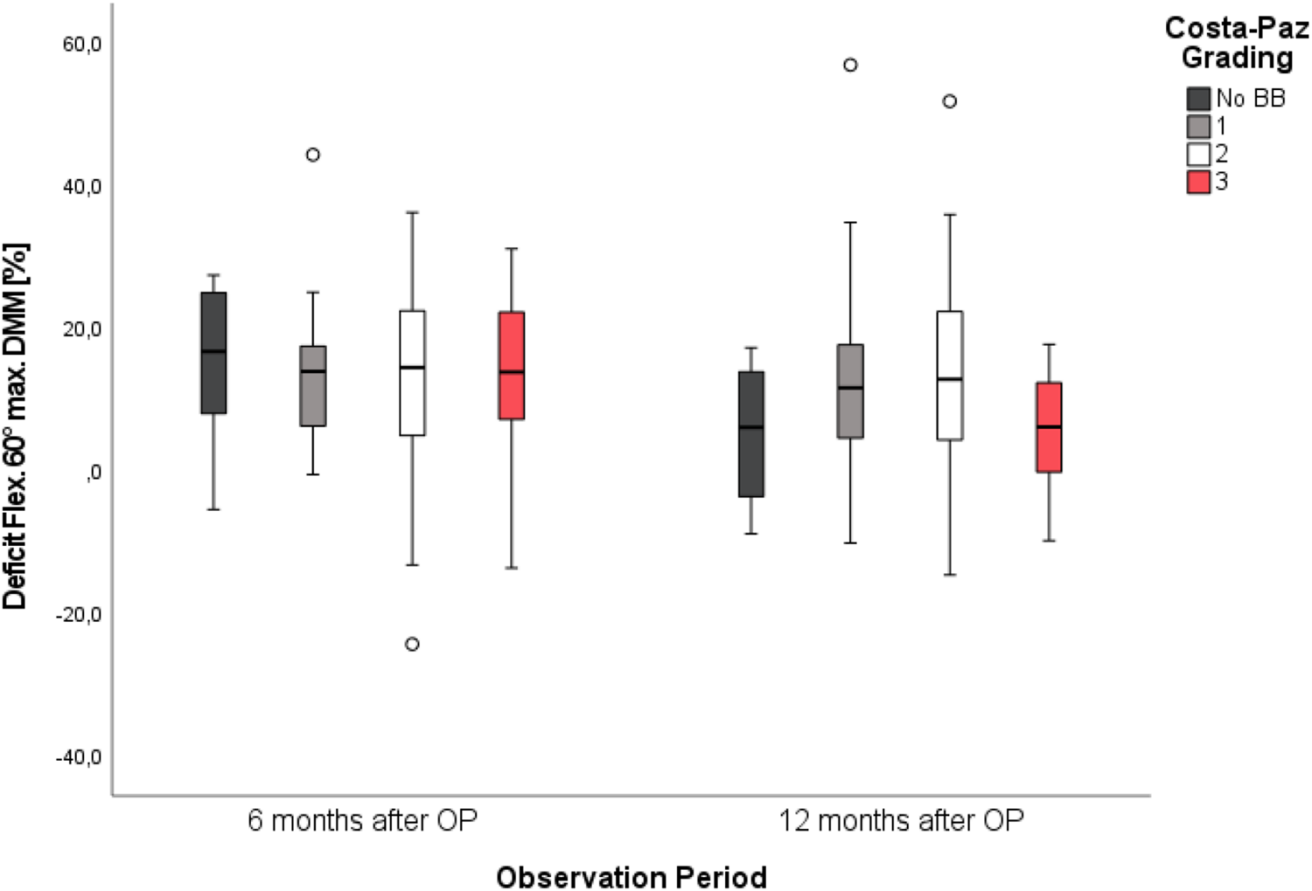
Isokinetic flexion strength improvement over time. Reduction of isokinetic strength deficit for knee flexion for all patients presenting with no BB and BB severities Costa-Paz 1–3 26 weeks (t2) and 52 weeks (t3) after the operation

Isokinetic testing (EXT_max_ [DMM], FLEX_max_ [DMM]) showed a significant reduction in strength deficit over time for both knee extension (F(2) = 29.96, p < 0.001) and knee flexion (F(2) = 6.67, p < 0.001), shown in Figs. 5 and 6. Neither the severity (Costa-Paz) nor the BB total volume had an impact on isokinetic muscle performance (n.s.). An additional medial BB distribution did not influence the results of isokinetic muscle performance significantly (n.s.).

## Discussion

The main goals of this study were to evaluate the influence of distribution pattern, severity and volume of BB in isolated ACL injuries on the functional outcome, quality of life and muscle strength following ACLR within the first year after surgery.

As the most important finding an impact of BB characteristics on short-term outcome measurements—including objective isokinetic testing—after ACLR for isolated ACL tears was disproved. The previously reported high prevalence and typical distribution pattern of BB could be confirmed by our results. Irrespective of BB morphology, all patients showed a significant improvement of subjective functional outcome, quality of life and muscle strength.

### Prevalence and distribution

Regarding the distribution pattern, the most common localizations mentioned in literature are the LTP and LFC with an overall BB prevalence up to 100% [3, 5–7, 10, 34]. Our data confirm that posttraumatic BB of LTP and LFC accompanying ACL tears can be expected regularly by radiologists and knee surgeons and should be interpreted as a safe secondary MRI sign of ACL injuries.

In a retrospective MRI study by Kim-Wang et al. after non-contact ACL tears and subsequent ACLR the authors found a posttraumatic BB in 135/136 patients [5]. Similar to the present study, the authors observed a high frequency of medial compartment bruising (72%) in contrast to earlier studies dealing with this issue. It is concluded that larger and more severe contusions—preferably occurring in the lateral compartment [34, 35]—may persist longer than less severe medial contusions. BB is known to resolve with increased time interval from injury [36, 37], while in a few cases it may persist on MRI taken up to one year after injury [38]. In the available literature investigating the prevalence of posttraumatic BB in ACL injuries and its impact on knee function, maximum time intervals between injury and MRI from 6 to 12 weeks were applied [5, 16], as in the present study the maximum interval is set to 12 weeks. Unfortunately, even in high-quality studies dealing with this issue the intervals are not specified [7].

Posttraumatic BB in the posteromedial tibial plateau is suspected to be a secondary sign of concomitant injuries like ramp lesions [39, 40] and collateral ligament injuries [41, 42]. In a recent study evaluating MRI of ACL-injured patients after ACLR Kim et al. found a correlation of BB localization in the LTP and MTP with the prevalence of lateral and medial meniscal tears [6]. Concomitant injuries were excluded in the present investigation. Nevertheless, all BB localizations were observed frequently in our study collective. It might be hypothesized that the correlation between BB localization and concomitant injuries should not be overestimated.

Recently, Korthaus et al. found an association of LFNS and ABF with lateral meniscal injuries and collateral ligament injuries [43]. This could not be verified in our study, as patients with accompanying injuries have been excluded in advance. Though, LFNS and ABF were present in 15.2%. According to literature, the proof of LFNS and ABF can be associated with inferior functional outcomes due to additional rotatory laxity after ACLR [27, 44] in the mid- to long-term follow-up. As this was not the key issue of this short-term investigation on isolated ACL tears, the impact of LFNS and ABF on functional outcome parameters should be evaluated in studies with longer follow-up periods.

### Injury mechanism

Several authors identified an association between BB distribution and injury mechanism, e.g., non-contact valgus injuries represented by BB predominantly in the LTP and LFC [3, 9, 34, 45–47]. Other authors found medial BB representative of extension and anterior tibial translation mechanisms [37, 48]. Zhang et al. performed a systematic review on BB associated with ACL injuries as indicators of injury mechanisms [46]. 12 studies with 589 patients were selected for review. The most common localization was LTP. The authors conclude that anterior tibial translation and maximal knee valgus occurring afterwards may be a primary injury mechanism in contact as well as non-contact ACL injuries.

Most patients included in the present study sustained non-contact ACL injuries (87.7%). Due to the study design, more detailed injury mechanisms could not be assessed. As the most common distribution patterns were LTP and LFC, it can be assumed that most patients had valgus-driven injury mechanisms.

### Influence of BB on outcome parameters

The impact of BB on functional outcome and quality of life after ACL injury and subsequent ACLR has been discussed controversially. Due to contrary study results its significance remains unclear.

Up to now, isokinetic strength testing has not been investigated regarding the impact of posttraumatic BB in ACL injuries. Isokinetic testing has a good to high test–retest reliability for patients after ACLR [49, 50] and is sensitive regarding the detection of subtle differences in torque production [33, 49–53]. In the recent study it was implemented for the first time to obtain an additional objective functional outcome parameter. It was hypothesized that muscle strength performance may be deteriorated by pain and functional deficits due to posttraumatic BB or subsequent early chondral wear in the affected regions.

Dunn et al. examined a large number of patients (n = 525) as part of a prospective cohort study (MOON) with regard to preoperative factors (including BB) associated with knee dysfunction and quality of life in the short-term at the time of index ACLR [12]. The authors could not identify an influence of BB localization on KOOS and SF-36 scores. Panjwani et al. reported on 270 patients who underwent ACLR within three months after ACL injury, evaluating the role of concomitant injuries and BB for preoperative knee function [14]. The authors found no influence neither of the presence of meniscal or chondral injuries nor of BB on preoperative KOOS and SF-36 scores. Similar results regarding the impact of BB on postoperative outcome of patients after ACLR are summarized in a systematic review by Walczak et al. [8]. The authors conclude that the presence of BB does not appear to significantly adversely affect the clinical outcome (IKDC) and quality of life (SF-36) of patients that underwent ACLR.

The results reported by Dunn, Panjwani and Walczak et al. are strongly supported by the findings of our study, as prevalence, distribution and severity of BB had no impact on functional outcome parameters and quality of life.

In contrast to these results, in 2017 Filardo et al. published a systematic review on BB associated with ACL injury, including 83 papers with a total of 10,047 patients [1]. The study group found a correlation of BB and higher pain levels and knee laxity after ACLR, if BB had larger volumes or medial compartment distribution. In a recent study by Agostinone et al. these findings are supported, reporting a correlation between intraoperative pivot-shift and BB severity of on the MTP [16]. Contrary to this, we could not reveal an influence of a medial BB distribution (MTP and/or MFC) or more severe BB (Costa-Paz II–III).

### BB volume measurement

Up to now, very few studies focused on BB volume as a predictor for the short- and mid-term outcome following ACLR [1, 7, 11, 15]. The present investigation is the first study including a separation of volumes by different localizations. Furthermore, volumes were quantified semi-automatically via software-assisted volumetry to objectify the obtained results.

Most authors could not demonstrate inferior postoperative results depending on the BB volume as an independent parameter [7, 11], as shown equivalently in the present study. The mean single volumes of BB, separated by the four different localizations, showed a similar order regarding its frequency like the distribution pattern: the most common localization (LTP) had also the highest mean volume (14.31 ± 9.93 cm^3^) in the collective, followed by LFC/MTP/MFC. This implicates that distribution pattern and single mean volumes can be used as equivalent parameters in the morphologic evaluation of posttraumatic BB after ACL injuries.

A correlation between BB volume and the interval injury—MRI was not demonstrated in this study. Thus, a “natural course” of BB regarding a dependency of volume from the time elapsed may not be present, as the healing time frame is quite variable [54].

### ACL injury and chondral damage

Initial macroscopic chondral damage is known to deteriorate the functional outcome after ACL injury and subsequent ACLR. In this context, Lattermann et al. reported on a cohort of 81 patients who underwent ACLR for primary ACL injuries [7]. The authors conducted a 2-/6-year follow-up, evaluating the influence of BB characteristics and initial concomitant articular pathologies on the functional outcome. Although BB severity and volume itself were not associated with inferior outcomes, cases with BB in combination with initial macroscopic chondral lesions were 3.4 more likely to have lower KOOS and IKDC scores following ACLR 6 years postoperatively. As concluded by Walczak et al., concomitant cartilage injury at the time of index ACLR may be one of the most important associated factors leading to inferior functional outcomes [8]. Macroscopic cartilage damage suitable for cartilage repair—ruled out by diagnostic arthroscopy at the time of ACLR—was an exclusion criterion in this study to investigate the role of BB alone in isolated ACL injuries, which may have led to the lack of significant impact of BB characteristics in this study.

Histological studies reveal a correlation between BB and certain tissue alterations, including microfracture of the subarticular spongiosa, osteocyte and chondrocyte necrosis in the adjacent joint area [55–57]. It can be hypothesized that the finding of BB associated with isolated ACL injuries may be accompanied by initially occult chondral lesions, leading to functional deficits and accelerate the progression of posttraumatic arthritis [7, 20, 24, 58].

As we found no detectable impact on the outcome parameters in the short-term follow-up, posttraumatic changes of the joint surfaces and subsequent arthritis may still be subclinical in the first year after ACLR but might reasonably become clinically relevant in the mid-term to long-term follow-up.

A recent MRI study on 40 patients after ACLR found a significant correlation between size of the initial BB in the MTP, LTP and LFC and increasing chondral wear in a 5-year follow-up [21]. In an earlier cohort study Potter et al. evaluated morphologic and clinical effects of BB after ACL tears, including n = 42 patients [20]. The size of lateral BB (LTP, LFC) in MRI was significantly associated with increased cartilage loss at follow-up examinations already 1 to 2 years after ACL injury. Though, a significant decrease in IKDC scores could not be demonstrated within the first 2 years but was shown in the mid-term follow-up 5 to 7 years post trauma. These study results implicate a discrepancy between the proof of early posttraumatic cartilage loss in MRI and a lack of clinical impact of BB within the first 2 years after ACL injury. On the other hand, BB gains more influence after 5 years as a significant decrease in IKDC scores could then be verified.

This conclusion is strongly supported by our study results, demonstrating a high prevalence of BB (91.8%) accompanying isolated ACL tears, 73.0% grade II–III BB according to Costa-Paz and high total BB volumes (max 57.32 cm^3^), but no influence on LS, TAS, IKDC, SF-36 and isokinetic muscle performance in the short-term follow-up.

### Limitations

This study has some limitations. Although functional outcome parameters were collected prospectively in context with a randomized trial, MRI analysis was performed in a retrospective manner. On the other hand, a homogenous cohort of n = 122 patients after ACLR for isolated ACL injury with complete data set was available. The intervals injury—MRI and injury—ACL reconstruction show a relatively wide range but refer to previous studies dealing with this topic. BB volume was measured quantitatively by software-assisted volumetry—but only in a subgroup of patients. Finally, the short-term follow-up 1 year after ACLR is a limitation of this study, as structural changes associated with the progression of posttraumatic arthritis may only become relevant in mid- to long-term analyses revealing a meaningful impact of BB characteristics.

## Conclusions

A negative impact of BB characteristics after ACLR on early functional outcome, quality of life and objective isokinetic strength could not be proved, as concomitant injuries were excluded systematically in this investigation. These findings may help the surgeon in preoperative counselling their patients and therapeutic decision making in ACLR. Even extensive BB findings in isolated ACL tears seem to have a limited implication for the timing of surgery and rehabilitation. Available data for prevalence and distribution of posttraumatic BB in ACL injuries could be confirmed. Thus, radiologists and knee surgeons can expect an accompanying BB with preferably lateral distribution pattern in isolated ACL tears regularly. A possible negative impact of distribution, severity and volume of BB can be hypothesized in the mid- to long-term follow-up after ACLR due to secondary arthritic changes, caused by initially occult chondral damage. Future research on a similar cohort after ACLR for isolated ACL tears, exclusion of concomitant injuries and longer follow-up periods is mandatory.

## Data Availability

The data used in this study is available from the corresponding author upon reasonable request.
